# New Insights on Molecular Mechanism of Hepatitis B Virus Covalently Closed Circular DNA Formation

**DOI:** 10.3390/cells9112430

**Published:** 2020-11-06

**Authors:** Alexander L. Marchetti, Haitao Guo

**Affiliations:** 1 Department of Microbiology and Immunology, School of Medicine, Indiana University, Indianapolis, IN 46202, USA; marchett@iu.edu; 2 Cancer Virology Program, Hillman Cancer Center, School of Medicine, University of Pittsburgh, Pittsburgh, PA 15213, USA; 3 Department of Microbiology and Molecular Genetics, University of Pittsburgh, Pittsburgh, PA 15213, USA

**Keywords:** hepatitis B virus, replication, covalently closed circular DNA, DNA repair

## Abstract

The chronic factor of the Hepatitis B Virus (HBV), specifically the covalently closed circular DNA (cccDNA), is a highly stable and active viral episomal genome established in the livers of chronic hepatitis B patients as a constant source of disease. Being able to target and eliminate cccDNA is the end goal for a genuine cure for HBV. Yet how HBV cccDNA is formed from the viral genomic relaxed circular DNA (rcDNA) and by what host factors had been long-standing research questions. It is generally acknowledged that HBV hijacks cellular functions to turn the open circular DNA conformation of rcDNA into cccDNA through DNA repair mechanisms. With great efforts from the HBV research community, there have been several recent leaps in our understanding of cccDNA formation. It is our goal in this review to analyze the recent reports showing evidence of cellular factor’s involvement in the molecular pathway of cccDNA biosynthesis.

## 1. Introduction

The hepatitis B virus (HBV) infection continues to be a global health burden with nearly 300 million people living with chronic hepatitis B (CHB), the primary disease causing around a million HBV-related deaths annually [1]. The horizontal transmission of HBV is achieved, for the most part, through sexual contact, the use of contaminated syringes, or contaminated blood transfusion. The disease in this context is, for the most part, realized through an acute self-limiting infection with approximately 5% of individuals progressing to chronic infection [2,3]. Vertical transmission, in stark contrast, is the major source of CHB carriers, who will likely live with the disease their entire lives and without treatment will in all likelihood succumb to the disease and its complications, including fulminant hepatitis, cirrhosis, and hepatocellular carcinoma [2,4,5,6].

**Figure 1:**
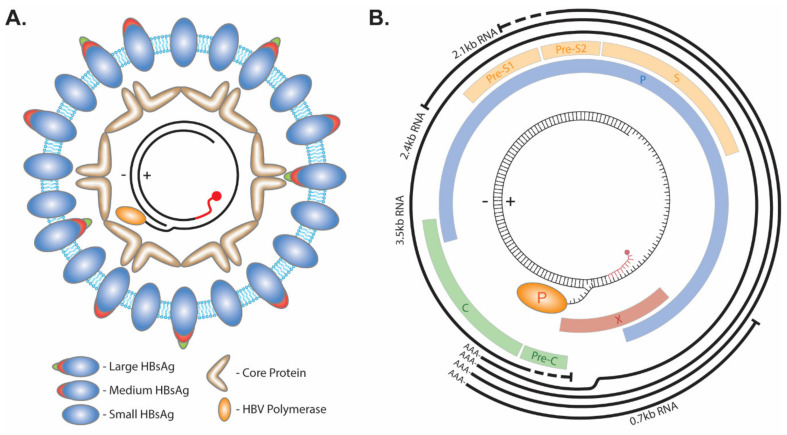
HBV structure and genome. (**A**) The envelope of HBV virion consists of lipids and the large, medium, and small viral surface proteins. Within the lumen of the virus, core protein forms an icosahedral capsid around the rcDNA genome which has the viral polymerase covalently attached to the 5′ end of the (−) strand and a capped RNA primer (red) attached to the 5′ end of the (+) strand. (**B**) The 3.2 kb HBV genome encodes four overlapping ORFs (Pre-Core/Core (Pre-C/C), Polymerase (P), Pre-S1/Pre-S2/S, and X) and four mRNA transcripts with an overlapping 3′ end (3.5 kb, 2.4 kb, 2.1 kb, and 0.7 kb).

The infectious HBV particle consists of a lipid bilayer membrane enveloped virus with three isoforms of the HBV surface protein: Large, Medium, and Small (Figure 1A). Within the envelope lies the HBV capsid made up of the HBV core protein. The capsid contains a partial double-stranded HBV genome called relaxed circular DNA (rcDNA). This form of the virus genome is made up by a short cohesive overlap between the 5′ ends of the two DNA strands. The minus (−) strand DNA molecule with a redundant portion on each terminal end results in an overhang flap of around eight nucleotides long. It is currently unknown whether the 3′ or 5′ end forms the flap. The viral polymerase is covalently attached to the 5′ end of the (−) strand via a tyrosyl-DNA phosphodiester bond. The plus(+) strand is rarely completed, with a gap ranging hundreds of nucleotides wide between the 3′ and 5′ end, and the latter is occupied by a capped RNA primer as a remnant of HBV reverse transcription [7,8,9]. The repair of these terminal peculiarities is essential for the progression of the virus life cycle, as they prevent HBV mRNA transcription (Figure 1B).

**Figure 2:**
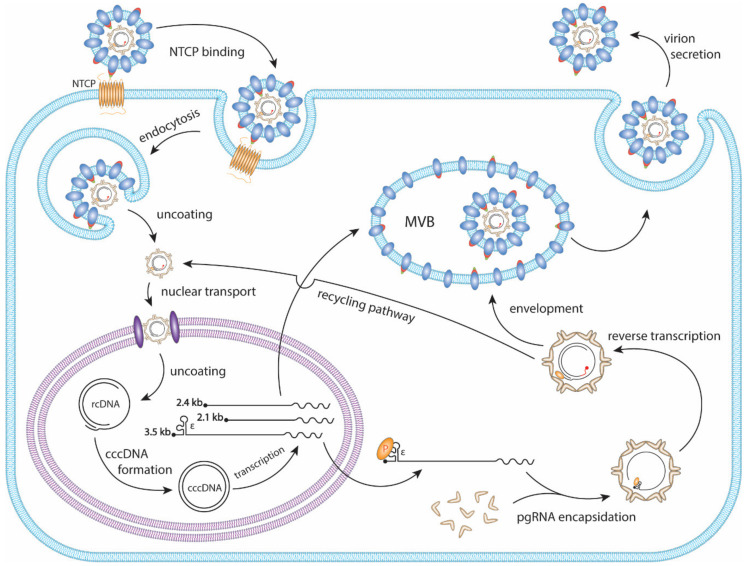
The HBV replication cycle. The HBV virion binds to the hepatic NTCP protein and is endocytosed. The viral membrane then fuses with the endosome, releasing the capsid into the cytoplasm, followed by nuclear import. The rcDNA is released into the nucleus where it uses host DNA repair mechanisms to form cccDNA. Viral mRNAs are transcribed from the cccDNA and are translated to viral proteins. In the cytoplasm, the viral polymerase binds to the epsilon (ε) structure of pgRNA and is encapsidated by core proteins. Within the nucleocapsid the pgRNA undergoes reverse transcription to form rcDNA. The mature capsid is enveloped and secreted as a virion particle through the MVB secretory pathway. The mature capsid can also be shuttled to the nucleus where it will amplify cccDNA through a process termed the rcDNA recycling pathway.

**Figure 3:**
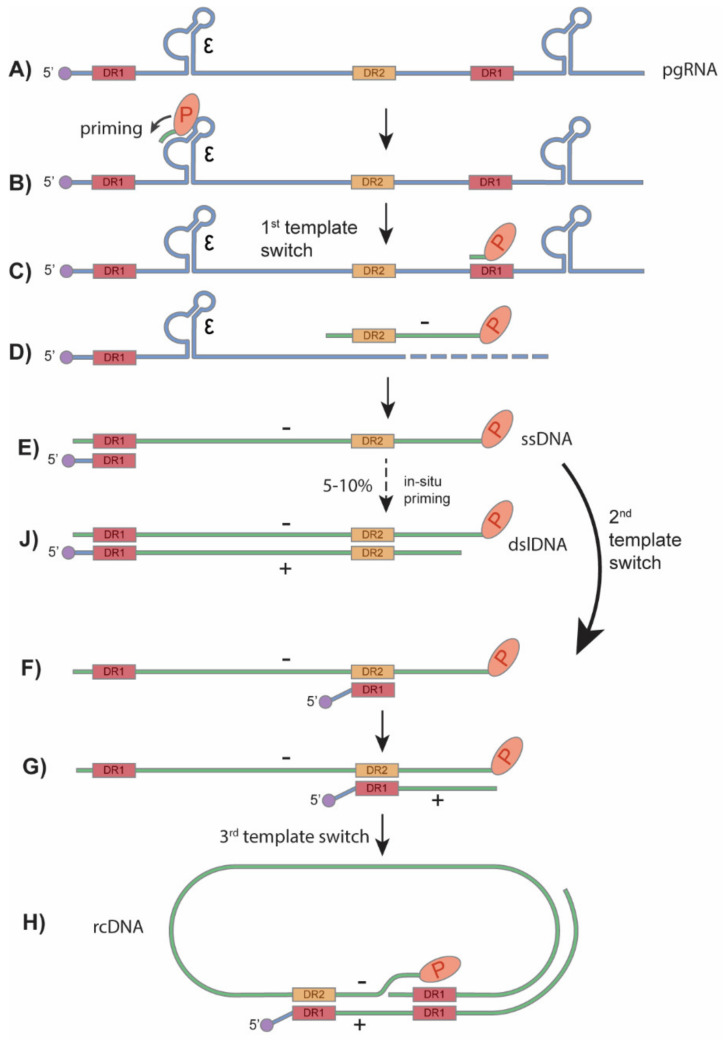
HBV rcDNA and dslDNA synthesis. (**A**) The encapsidated pgRNA is the template for HBV reverse transcription. (**B**) Inside of viral capsid, HBV polymerase binds to the ε structure of the pgRNA and begins priming with 3 nucleotides. (**C**) The first template switch occurs and the polymerase along with the covalently attached primed DNA move to the direct repeat 1 (DR1) motif at the 3′ end of the pgRNA. (**D**,**E**) The polymerase continues to synthesize the (−) strand DNA and digest the read RNA until it reaches the 5′ end of the pgRNA. (**F**) The second template switch occurs when the uncleaved 5′ DR1 region of pgRNA moves to the DR2 region of the newly synthesized (−) strand DNA. (**G**,**H**) Plus strand DNA synthesis then begins and undergoes a 3rd template switch to the 3′ end of the (−) strand, circularizing the double-stranded DNA and forming the rcDNA. (**J**) At a low frequency, the second template switch fails to occur, and the RNA primer undergoes an in situ priming to synthesize the HBV dslDNA. Adapted from Flint et al. [41].

HBV primarily infects hepatocytes. The infection begins with low-affinity binding of the virion particle to the heparan sulfate proteoglycans (HSPG) [10]. Upon the binding of the pre-S1 domain of viral large surface protein to the liver-specific receptor, sodium taurocholate co-transporting polypeptide (NTCP), the virion is endocytosed [11,12,13,14] (Figure 2). Within the endosome the viral particle fuses to the endosomal membrane, releasing the viral capsid into the cytoplasm, where it unfolds to expose the nuclear localization signal (NLS) of the core protein [15,16,17]. It is then, in turn, brought to the nuclear pore complex where the rcDNA is imported into the nucleus [18,19,20]. The rcDNA undergoes several enzymatic steps to repair its terminal peculiarities, resulting in the formation of covalently closed circular DNA (cccDNA). Following its formation, cccDNA proceeds to transcribe its 3′-end overlapping mRNAs, including the 3.5 kb precore mRNA and pregenomic RNA (pgRNA), 2.4 kb and 2.1 kb surface mRNA, and 0.7 kb X mRNA (Figure 1B and Figure 2). In the cytoplasm, pgRNA translates viral core proteins and polymerase. The viral polymerase binds to the epsilon structure on the pgRNA and is encapsidated. Within the viral capsid, the viral polymerase begins to reverse transcribe the pgRNA into the (−) strand DNA. The reverse transcription is initiated via polymerase’s protein priming activity against the epsilon structure, resulting in the covalent attachment of polymerase to (−) strand DNA. Next, three sequential template switches take place to produce rcDNA: (1) the Polymerase-primed nascent DNA translocates to direct repeat (DR) 1 at the 3′ end of pgRNA to continue the full-length (−) strand DNA synthesis, and the pgRNA template is degraded simultaneously by the RNase H activity of polymerase; (2) the undigested DR1-containing sequence at 5′ end of pgRNA translocates to DR2 at the 5′ end of (−) strand DNA and serves as a primer to start (+) strand DNA synthesis; (3) the elongation of (+) strand DNA rapidly reaches the 5′ end of (−) strand DNA, then switches over to the 3′ end of (−) stand DNA for further synthesis and thus circularizes the DNA into a rcDNA format (Figure 3). The 2nd template switch can fail to occur in a low frequency, resulting in an in situ priming of (+) strand DNA and a double-stranded linear DNA (dslDNA) product. During the (+) strand DNA synthesis, the capsid is enveloped at the multivesicular body (MVB) [21,22], and the nucleotide supply is perhaps exhausted prior to (+) strand completion [23]. The enveloped virus is then transported to the cell membrane and secreted as progeny virion. In addition, the cytoplasmic mature capsid can be redirected into the nucleus to supply more rcDNA for cccDNA formation, which is called intracellular rcDNA recycling or cccDNA amplification [7,23,24,25] (Figure 2).

Although the rcDNA in virion particles is infectious, it cannot replicate itself or express its open reading frames without “healing” these inhibitory terminal structures first. Therefore, to begin the viral replication process, it is essential to form its cccDNA. cccDNA, as its name implies, is the completed double-stranded circular form of the virus genome. While the dslDNA, a byproduct of rcDNA synthesis, can be converted into cccDNA or integrated into the host genome and still have active transcription for certain viral mRNAs, the only source of genuine novel infectious HBV particles is cccDNA expression [9,26,27,28]. Despite the virus’ complete reliance on cccDNA for reproduction, none of its proteins have been shown to have an essential role in rcDNA to cccDNA conversion in the nucleus [24,25,29,30,31,32,33,34,35,36]. Instead, the virus relies heavily, if not completely, on nuclear host factors for converting rcDNA into cccDNA [9,37,38,39,40].

Currently, there are limited options for treatments of CHB and the most effective way to stop the spread of CHB continues to be vaccination programs [4]. There are two main methods of treating CHB: Pegylated IFN-alpha and Nucleos(t)ide analogues (NUCs). Pegylated IFN-alpha is an immunomodulatory treatment for a minority of CHB patients but is usually ceased after a year due to side effects and low responses; only approximately one-third of the treated patients respond to IFN-alpha treatment [42]. NUCs inhibit the viral polymerases and therefore the reverse-transcription step and are successful in reducing the viral titer in CHB patients, functionally curing them of a majority of the viral pathology. This, however, is not a true cure, as the cessation of treatment will most often result in a rapid viral rebound, requiring patients with CHB to be on NUC treatment for their entire lives, and the development of drug-resistance is not uncommon [43,44,45]. Nonetheless, NUCs are rarely able to completely clear the virus. This is because NUCs inhibit the virus during its reverse transcription step, which occurs after the establishment of cccDNA, and therefore have no or little effect on the first round of cccDNA formation during de novo infection [32,33,46,47]. Once cccDNA is established it will continuously express viral RNA and proteins throughout NUC treatment, which can still cause pathology without robust viral replication. Similarly, the integrated HBV DNA, which is unable to produce progeny virus, can be an additional source of pathology via altering host chromosome stability and expressing HBsAg [26]. Without targeting HBV cccDNA, the virus can maintain a reservoir of cccDNA in hepatocytes that will remain safe from the standard treatments [9,38,39]. It is, therefore, a consensus within the HBV research community that a genuine cure to HBV will have to involve targeting and eliminating cccDNA reservoirs [1,43]. Despite the necessity of targeting HBV cccDNA, very limited knowledge about cccDNA formation was available until several recent studies began to pull back the veil on the virus-host interactions in charge of this essential HBV life cycle step.

## 2. HBV cccDNA Formation

### 2.1. General Steps of cccDNA Formation

**Figure 4:**
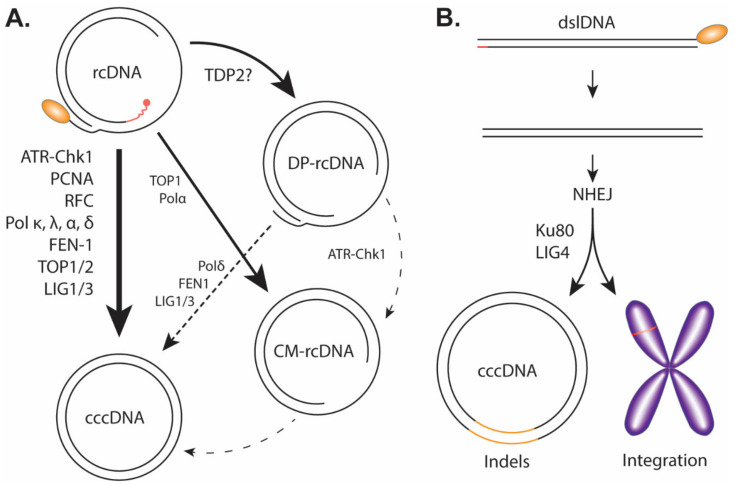
Proposed pathway of cccDNA formation. (**A**) Genuine cccDNA would be classified as cccDNA converted from rcDNA and would therefore have to undergo a relatively high-fidelity DNA repair. The host factors listed have been heavily implicated in the formation of genuine cccDNA. It is important to note the exact temporal/mechanistic relationship these factors have with cccDNA formation is yet to be fully explored or explained. DP-rcDNA and CM-rcDNA have been thoroughly shown to be downstream of rcDNA. Though there is credible evidence to suggest DP-rcDNA, or an unclassified subset of DP-rcDNA, is necessary for cccDNA formation, it remains uncertain if these populations including CM-rcDNA are actual genuine intermediates to cccDNA. The lines drawn between the HBV DNA populations correspond to the certainty (solid lines) and uncertainty (dotted lines) of their relationships, and the host DNA repair enzymes that have been implicated in each DNA transitions are indicated. (**B**) Through non-homologous end joining (NHEJ) mechanism, the HBV dslDNA can be integrated into the host genome or circularized to a pseudo cccDNA with substantial indels.

To kick off cccDNA formation, the cytoplasmic viral capsid containing the mature rcDNA, either from the incoming virus or the de novo viral DNA replication, needs to prepare for the nuclear transportation via a conformational change to expose its C-terminal Domain (CTD) nuclear localization signals (NLS) for karyopherin binding [17,48,49]. The viral and host mechanism(s) regulating HBV capsid NLS exposure is unclear; however, previous studies suggest that the rcDNA maturation, deproteination, and/or the binding of karyopherin with capsid could be a trigger [17,50,51,52,53]. The entire capsid is then imported through the nuclear pore complex into the nucleus where it then releases the rcDNA into the karyoplasm [48,49]. From cytoplasm to the nucleus, HBV rcDNA must undergo a series of enzymatic reactions to repair/remove the previously described obstructive terminal peculiarities: the viral polymerase must be removed presumably by unlinking the tyrosyl-5′-DNA phosphodiester bond, RNA primer needs to be removed, (+) strand DNA needs to be completed, one copy of the terminal redundant sequence on the (−) strand DNA must be removed and, finally, the (+) and (−) strands must be ligated close to form cccDNA (Figure 4A).

### 2.2. cccDNA Intermediates/Precursors

The order in which these catalytic events occur is not completely understood. Due to the complexity of rcDNA to cccDNA conversion, it is expected that DNA intermediate(s) may exist during cccDNA formation.

*dsl-ltr-DNA*: More than two decades ago, a hypothetical linear hepadnavirus genome, termed double-stranded linear HBV DNA with long terminal repeats (dsl-ltr-DNA), which contains terminal duplication of the cohesive region (between DR1 and DR2) from rcDNA by displacement synthesis through the cohesive overlap, has been proposed to be a cccDNA precursor. Through homologous recombination of these LTRs, the dsl-ltr-DNA could bypass the removal of the terminal structures of rcDNA and form cccDNA directly [54]. However, it has not been directly detected and thus remains mysterious [54]. Instead, cccDNA species with extensive insertion/deletions (indels) have been discovered and would somewhat indicate that a dsl-ltr-DNA intermediate may occur but is transient and is ligated through non-homologous end joining (NHEJ). However, it is now well acknowledged that the viral dslDNA replicative intermediate is the predominant precursor for indels-containing cccDNA formation through NHEJ [24,55,56,57,58].

*DP-rcDNA (PF-rcDNA):* More than one decade ago, we and others systematically characterized a rcDNA species without the covalently attached viral polymerase, which was termed as deproteinized rcDNA (DP-rcDNA) also known as protein-free rcDNA (PF-rcDNA) (Figure 4A) [24,25]. It is worth noting that DP-rcDNA had shown up in even earlier studies but did not draw much attention at that time [59,60]. Deproteinated dslDNA (DP-dslDNA) also exists but protein-free ssDNA does not, and multiple reports indicate that deproteination occurs selectively on mature double-stranded viral DNA [17,24,25,57]. The DP-rcDNA can be extracted by Hirt DNA extraction method, which is also used to extract cccDNA [61,62]. In the absence of protease digestion, a phenol treatment during Hirt DNA extraction from HBV replicating cells allows for the polymerase covalently bound rcDNA to become soluble in the phenol fraction, leaving behind the DP-rcDNA and cccDNA as protein-free DNA. The cell fractionation showed a significant population of DP-rcDNA in the cytoplasm as well as the nucleus, suggesting that the rcDNA deproteination step occurs prior to nuclear import [25]. Further studies on cytoplasmic DP-rcDNA suggested that the completion of viral (+) strand DNA inside the nucleocapsid triggers rcDNA deproteination and nucleocapsid conformational shift, resulting in the exposure of the nuclear localization signals (NLS) on the C-terminus of capsid protein, followed by binding of karyopherins and nuclear import of DP-rcDNA containing capsid [17]. The conformational change or partial disassembly of cytoplasmic DP-rcDNA-containing capsid was also inferred by the accessibility of encapsidated DP-rcDNA by DNase I [17,25]. In line with this, another study reported that DP-rcDNA was predominantly found in nucleus, which was likely due to the treatment of cytoplasm samples with Turbonuclease before Hirt DNA extraction [24]. Further analyses of the cytoplasmic DP-rcDNA demonstrated that the (+) strand DNA is complete or almost complete with the RNA primer being removed from the 5′ end, and the viral polymerase is completely removed from the 5′ end of (−) strand DNA through unlinking the tyrosyl-DNA phosphodiester bond with the terminal redundant sequence remaining on both ends (Figure 4A) [63]. In the nucleus, DP-rcDNA is released from the capsid and converted into cccDNA by employing the host DNA repair machinery [17,25,57,64].

The existing evidence supporting DP-rcDNA as a functional precursor of cccDNA includes but may not be limited to: (1) it always appears earlier than cccDNA in HBV-transfected or -infected cells [24,25,47,65,66]; (2) inhibition of rcDNA deproteination by compounds or blocking DP-rcDNA nuclear transportation resulted in the accumulation of cytoplasmic DP-rcDNA but a reduction of nuclear DP-rcDNA and cccDNA [17,67]; (3) inhibition of non-homologous end joining (NHEJ) DNA repair pathway in cells exclusively replicating duck HBV (DHBV) dslDNA genome resulted in accumulation of nuclear DP-dslDNA but reduction of cccDNA [57]; (4) transfection of purified DP-rcDNA into cells resulted in viral DNA replication, suggesting a successful conversion of DP-rcDNA into cccDNA [25]. Nevertheless, whether DP-rcDNA is the major precursor for cccDNA remains uncertain. In the HBV stably transfected cells, such as HepG2.2.15, HepAD38 cells and HepDE(S)19 cells, that support cccDNA formation exclusively through the intracellular amplification route, nuclear DP-rcDNA normally accumulates to a much higher level than cccDNA [24,25,59,64,67,68], indicating that the majority of nuclear DP-rcDNA may be a dead-end product or there is a rate-limiting mechanism for converting DP-rcDNA into cccDNA. However, the levels of DP-rcDNA are similar to or even less than cccDNA in HBV-infected cells in vitro and in vivo [35,66,69,70,71,72], indicating that the production, role, or conversion efficiency of DP-rcDNA in cccDNA formation may be different between HBV transfection and infection systems. The DHBV system is helpful in the study of HBV cccDNA formation as the viruses are closely related and therefore have similar genomes and lifecycles [40]. One major advantage is that the DHBV model produces more cccDNA than HBV even in transfected human hepatocyte-derived cells, in which HBV cccDNA is often difficult to detect due to low copy numbers [58,64]. Previous studies using DHBV system have identified similar DP-rcDNA intermediate and certain host DNA repair factors shared by HBV in cccDNA formation [17,24,25,57,58]. However, it is worth noting that the robust cccDNA formation capacity of DHBV through the rcDNA recycling pathway is likely dependent upon a virus-specific mechanism(s) [64], thus there may be different regulations at the early steps of cccDNA formation between DHBV and HBV.

*CM-rcDNA:* A recent study has reported observing another possible cccDNA intermediate believed to be a closed (−) strand rcDNA (CM-rcDNA) population (Figure 4A) [73]. In this study, by treating Hirt DNA extraction samples with exonucleases Exo I/III that digest non-circular DNA with unblocked 3′ end, the DP-rcDNA was removed but cccDNA was preserved, and a minor DNA species of (−) polarity was evident on the Southern blot below single-stranded linear DNA position. This minor species was identified as a single-stranded circular DNA derived from the CM-rcDNA by removing the open circular (+) strand DNA through Exo I/III digestion. The absence of single-stranded circular (+) DNA upon Exo I/III treatment of Hirt DNA implied that the circular (−) DNA was not a result of randomly nicked cccDNA which would have resulted in two equally sized populations. The presence of CM-rcDNA indicates that the peculiarities on the (−) strand DNA are repaired and the termini are ligated earlier than (+) strand of rcDNA during cccDNA formation, though it remains unclear whether CM-rcDNA is a genuine precursor for cccDNA or a byproduct during cccDNA formation. It is also unknown whether CM-rcDNA is a derivative of the aforementioned DP-rcDNA or co-produced together with DP-rcDNA from rcDNA.

### 2.3. DNA Repair Factors Involved in cccDNA Formation

Once the rcDNA is delivered in the nucleus and uncoated, it is expected that the structural peculiarities on the termini of rcDNA will be recognized as DNA damage signals by the host DNA repair apparatus and converted into cccDNA [9,37,39,74]. Through a variety of experimental approaches, a handful of cellular DNA repair factors involved in cccDNA formation have been identified as follows.

*ATR-Chk1:* There are two major high-fidelity cellular DNA damage response (DDR) pathways, the ataxia telangiectasia mutated (ATM) pathway and the ataxia telangiectasia and Rad3-related (ATR) pathway, which are two distinct kinase signaling cascades leading to DNA damage checkpoints [75]. To assess whether DDR is involved in rcDNA to cccDNA conversion, Luo et al. treated HBV-infected HepG2-NTCP cells and primary human hepatocytes with ATR and Chk1 inhibitors (ATR/Chk1 inhibitors: AZD6738, VE-821, and CHIR-124) and observed a marked decrease in cccDNA levels, but not under ATM inhibitor treatment (ATM inhibitors: KU-55933 and KU-60019) [76]. HBV stable cell lines AML12HBV10 and HepAD38, which support cccDNA formation through the rcDNA recycling pathway, also illustrated a similar inhibition of cccDNA production when treated with the ATR-Chk1 inhibitors or siRNA. Thus, the role of the ATR-Chk1 pathway is not limited to the initial round of cccDNA formation but also the internal cccDNA amplification. Considering that ATM-Chk2 and ATR-Chk1 pathways are activated by double-strand breaks and persistent single-stranded DNA [77], the involvement of ATR-Chk1 in cccDNA formation is likely due to the single-strand gaps on rcDNA. Interestingly, the study found that PF-rcDNA (aka DP-rcDNA) levels increased under the ATR-Chk1 inhibitor conditions, but CM-rcDNA was reduced to a similar degree of cccDNA, indicating that CM-rcDNA may be processed from DP-rcDNA by ATR-Chk1 machinery. Under the condition of ATR-Chk1 inhibition, a smear also appeared between the DP-rcDNA and cccDNA bands on Southern blot, which were determined to be the DP-rcDNA intermediates lacking large portions of their 5′ end of (−) strand DNA, indicating that ATR-Chk1 pathway may protect the deproteinated rcDNA from cellular nuclease digestion.

*DNA Polymerases:* It has been reported that inhibiting hepadnavirus polymerase by NUCs did not block the first round cccDNA formation in in vitro virus infection, indicating that host polymerase(s) are responsible for repairing rcDNA into cccDNA [32,33]. In one study, a siRNA knockdown screen of 15 cellular polymerases in HepG2-NTCP cells was carried out. HBV 3.5kb RNA and HBeAg were used as markers of cccDNA formation and successful infection. The knockdown of Pol κ, Pol η, or Pol λ significantly reduced both 3.5 kb RNA and HBeAg, with Pol κ knockdown having the most significant reduction [35]. Co-inhibition of Pol κ and other polymerases showed enhanced inhibition of infection, but Pol κ was observed to have the most critical role. Pol κ was knocked out using the CRISPR/Cas9 system in HepG2-NTCP cells. Infection assays were performed on the Pol κ knockout cells and cccDNA was observed through a Southern blot. There was no observable cccDNA band in the −/− Pol κ cells and a reduced band in the +/− Pol κ cells. A knockout of Pol λ in HepG2-NTCP cells was also made using similar methods and was also observed through Southern blotting to reduce cccDNA formation. While not as significant of a reduction as the Pol κ knockout, Pol λ was still shown to have a role in cccDNA formation. Pol κ, of the Pol Y family, is known for its role in trans-lesion DNA synthesis and the nucleotide excision repair (NER) pathway [78]. Although the exact mechanism of its involvement was left unexplored, it is implied by Pol κ’s function that it is perhaps involved in the completion of (+) strand DNA of nuclear rcDNA.

In another study, Tang et al. investigated the role of polymerase alpha in cccDNA formation and uncovered its crucial role in cccDNA formation through the rcDNA recycling pathway [79]. Phosphonoformic acid (PFA), a reversible HBV polymerase inhibitor, was used to arrest HBV replication in HepAD38 cells. Once PFA is removed, there is synchronized mass production of cccDNA from newly synthesized rcDNA, allowing for transient treatment of potential toxic inhibitors of cellular polymerases. Pol B family polymerases α, δ, and ε were inhibited using pan-inhibitor Aphidicolin (APH). This resulted in a marked decrease in cccDNA formation. To investigate the individual polymerases further, a CRISPR/Cas9 knockout of Pol δ was made in HepAD38 cells. The knockout caused a modest decrease in cccDNA formation, however, when paired with APH there was a marked decrease in cccDNA levels. Although Pol κ is involved in de novo cccDNA formation as mentioned above [35], these results suggest that it is not the main effector in the intracellular cccDNA amplification pathway. The role of Pol α in cccDNA formation was further inspected by specifically inhibiting it using CD437. The specific inhibition of Pol α showed a large decrease in cccDNA formation without changing cccDNA stability, together with a co-reduction of CM-rcDNA, indicating that Pol α may be involved in the process of rcDNA (−) strand ligation. The requirement for different cellular polymerases in de novo cccDNA formation and the intracellular cccDNA amplification is less understood but may be related to the potentially different structures of enveloped capsid compared to intracellular naked capsid or their rcDNA content. Pol B family polymerases are the primary subunits of cellular DNA replication and are most active during the S phase of replicating cells [80]. This could suggest that the intracellular cccDNA amplification, through the rcDNA recycling pathway, may occur specifically in dividing cells, lending a plausible explanation of how cccDNA reservoirs are conserved amid mitosis.

*FEN1:* When rcDNA or DP-rcDNA is imported into the nucleus, one copy of the terminal redundancies must be removed before the ligation of the (−) strand. Flap endonuclease 1 (FEN1) is a eukaryotic 5′-flap endonuclease involved in DNA replication and repair, and its major function is to remove the 5′-flap structure of Okazaki fragments during lagging strand DNA synthesis [81]. In this regard, the terminal redundant sequences at both ends of the rcDNA (−) strand represent the junction of two Okazaki fragments if a 5′-flap is formed (Figure 1B and Figure 4A). Kitamura et al. performed an in vitro FEN1 activity assay using a synthetic DNA substrate mimicking a putative 5′-flap structure of the terminal redundancy of HBV rcDNA and demonstrated FEN1′s ability to bind and cleave the synthetic HBV rcDNA 5′-flap [82]. Two catalytically defective FEN1 proteins were unable to cleave the 5′-flap and a FEN1 inhibitor, 3-hydroxy-5-methyl-1-phenylthieno[2,3-d]pyrimidine-2,4(1H,3H)-dione (PTPD), inhibited the synthetic HBV DNA cleavage. CRISPR/Cas9 knockdown and PTPD inhibition of FEN1 in HepAD38.7 cells reduced cccDNA levels, suggesting FEN1′s enzymatic activity is involved in the formation of cccDNA. The chemical inhibition of FEN1 through PTPD in HepG2-NTCP cells showed a consistent decrease in cccDNA formation in an infection model cell line. A FEN1-HBV ChIP qPCR confirmed FEN1 binds to HBV DNA in cells. The proposed role of FEN1 in removing the 5′ terminal redundancy of HBV rcDNA is consistent with a previous study indicating that the 5′ terminal redundancy of DHBV rcDNA is preferentially removed during cccDNA formation [83]. Considering that FEN1 recognizes the free 5′-end of the flap and threads the ssDNA strand through its helical arch and enzymatic site to create a configuration for cleavage [81], this threading requirement suggests that FEN1 cannot directly cleave the rcDNA with polymerase attached, and DP-rcDNA can serve as the substrate for FEN1. However, since a complete inhibition of HBV cccDNA formation was not achieved through FEN1 inhibition, it remains unknown whether other 5′-flap endonucleases, including Dna2 and XPG, or even exonucleases, are playing an overlapping role in cccDNA formation.

*DNA ligases:* Our previous study investigated possible DNA repair factors involved in cccDNA formation through a large shRNA screen of DNA repair related proteins in HepDES19 cells [58]. The screen revealed host ligases as potential host factors for cccDNA formation. This result was investigated further in an in vitro cell-free cccDNA formation assay. DHBV rcDNA was collected and incubated with nuclear extracts and cccDNA formation was measured through a sensitive PCR assay. When the system was treated with ligase inhibitors, the cccDNA amplicon was completely blocked, suggesting the ligases were involved in forming the cccDNA. The pan ligase inhibitor, L189, inhibited DHBV cccDNA formation in a DHBV inducible cell line, HepDG10, consistent with the results of the in vitro assay. Specific knockouts of LIG1 and LIG3 were made with CRISPR/Cas9 in HepDG10 cells. The depletion of LIG1 and LIG3 significantly reduced cccDNA formation but did not seem to affect rcDNA levels, suggesting they have a specific role in cccDNA formation. Similar knockouts of LIG1 and LIG3 were made in the HBV inducible cell line, HepDES19, and showed a similar reduction in cccDNA formation. This phenotype was also replicated in infection assays with HepG2-NTCP cells when LIG1 and LIG3 were individually knocked down by shRNA. Therefore, the study indicated that LIG1 and LIG3 play an overlapping role in HBV cccDNA formation, likely acting on the last step of rcDNA to cccDNA conversion by ligating the ligatable ends of rcDNA. It remains unknown whether LIG1 and LIG3 have DNA strand-specificity on rcDNA ligation, including the formation of CM-rcDNA. In addition, LIG4, a component of NHEJ pathway, has been shown to be responsible for noncanonical cccDNA formation from dslDNA [58].

*DNA topoisomerases:* Topoisomerase (TOP) 1 and 2 transiently nick/cut DNA to release torsion built up during DNA replication occurring in splitting cells or DNA repair [84], which were recently shown to be involved in cccDNA formation [85]. A PFA arrest and synchronized release in HepAD38 cells similar to that previously described was also used in this study to allow short-term treatment of normally toxic TOP inhibitors. Interestingly, inhibition of TOP1 by Topotecan or Camptothecin reduced cccDNA and CM-rcDNA levels. On the other hand, when TOP2 was inhibited with Idarubicin and Doxorubicin, it only affected cccDNA levels. This study suggests that TOP1 and 2 have potentially separate roles in cccDNA formation, which TOP1 is potentially involved in the repair/ligation of the rcDNA (−) strand during CM-rcDNA formation if CM-rcDNA is indeed a precursor for cccDNA. However, the detailed molecular mechanism underlying TOP1/2-mediated cccDNA formation remains elusive, including how and where TOP1/2 interact with rcDNA/cccDNA, how TOP1/2 cooperate with DNA ligases to close rcDNA, and whether other topoisomerases play overlapping roles in cccDNA formation.

*Tyrosyl-DNA phosphodiesterase 2 (TDP2):* At times topoisomerases can fail to release the cut and untwined DNA and remain covalently bonded to the strands. To avoid the potentially lethal consequences, tyrosyl-DNA phosphodiesterases are recruited to cleave the covalent phosphor-tyrosyl bond, allowing the DNA to be ligated shut [86,87]. Tyrosyl-DNA phosphodiesterase 2 (TDP2) specifically cleaves the 5′ bound TOP2 [88,89], which resembles the 5′ tyrosyl-DNA phosphodiester bond between HBV polymerase and rcDNA (Figure 1). It was for this similarity that researchers investigated TDP2′s potential to be involved in the removal of the viral polymerase from rcDNA and subsequently the formation of HBV cccDNA. The in vitro biochemical assays demonstrated that human TDP2 exhibited an ability to cleave the 5′-tryosyl bound DHBV and HBV Polymerases from newly primed nascent ssDNA or mature rcDNA [90,91,92,93]. A knockdown of TDP2 was then carried out with shRNAs in DHBV-transfected Huh7 cells. The result showed that a knockdown of TDP2 was associated with a delay in cccDNA formation, and ectopic expression of TDP2 in the TDP2 knockdown cells restored cccDNA formation kinetics [93]. This potential role of TDP2 in cccDNA formation, however, has been refuted by other studies. Cui et al. established a HepG2/NTCP TDP2 knockout cell line and observed the cells to be permissive to HBV infection [91]. The study also showed that TDP2 knockdown in HepAD38 via siRNA resulted in a modest increase of cccDNA, and overexpression of TDP2 resulted in a modest decrease in cccDNA formation in HBV transiently transfected cells. However, when a DHBV replication plasmid was transfected into TDP2 knockout HepG2 cells, the group observed a moderate decrease in DHBV cccDNA formation. Furthermore, the chemical inhibitors of TDP2 failed to inhibit HBV infection in a hepatic co-culture system [94]. Our recent study on mapping the termini of cytoplasmic DP-rcDNA suggested a role of tyrosyl-DNA phosphodiesterase in removing viral polymerase from rcDNA; however, the knockout of TDP2 in HepAD38 cells did not markedly reduce the levels of DP-rcDNA and cccDNA [63]. These observations cast doubt on whether TDP2 is essentially involved in the cleavage of the viral polymerase on rcDNA during the formation of DP-rcDNA and/or cccDNA, or there is a cellular compensatory activity from a TDP2-like protein in the absence of TDP2.

*Lagging strand synthesis machinery:* As above mentioned, the terminal structures on HBV rcDNA can resemble Okazaki fragments, the phenomena resulting from 5′ to 3′ DNA synthesis on the “lagging” strand DNA. It takes the coordination of multiple proteins to repair these fragments. Due to their similarity, one group recently investigated if the lagging strand synthesis machinery was involved in the repair of rcDNA [36]. First, a synthetic DNA substrate mimicking the structure and terminal modifications of rcDNA was created. When incubated with yeast and human cellular extracts, the synthetic recombinant rcDNA (RrcDNA) was able to form cccDNA. Five core components involved in lagging strand synthesis, including PCNA, RFC, FEN1, Pol δ, and LIG1, were then immunodepleted in yeast extracts, resulting in complete or significant inhibition of cccDNA formation. The absence of any one of these proteins completely inhibited the formation of cccDNA in vitro. This research corroborates and enhances the evidence showing certain DNA repair factors having important roles in cccDNA formation. While in vivo cell culture assessment did not show the complete loss of cccDNA compared to these yeast and hepatic cell extract in vitro assays, they still lend themselves to these host factors’ potentially crucial roles in the virus’s lifecycle.

*NHEJ:* While NHEJ is the most readily available form of DNA repair to host cells, it is unlikely the main pathway utilized by the virus to repair its rcDNA due to the error-prone propensity of NHEJ [95]. However, as previously mentioned, it has been reported to be an accessory pathway for cccDNA formation from aberrant or unusual precursors. Precursors such as dsl-ltr-DNA and dslDNA can be converted into cccDNA utilizing NHEJ mechanism [27,54,55,56]. Previous studies have explicitly demonstrated the essential role of NHEJ core components, including Ku80 and LIG4, in DHBV cccDNA formation from viral dslDNA precursor (Figure 4B) [57,58]. Insertions and deletions (indels) between DR1 and DR2 are common in this DNA repair pathway and have been observed in dslDNA-derived cccDNA [24,55,58]. It is therefore a dead end for rcDNA replication purposes due to the gross indels, though illegitimate dslDNA replication may still occur via subsequent rounds of NHEJ-mediated cccDNA formation from the newly synthesized dslDNA mutants [55]. In addition, NHEJ is also responsible for dslDNA integration into the host chromosomes at the double-strand break sites (Figure 4B) [27,96].

### 2.4. Non-DNA Repair Factors Involved in cccDNA Formation

DNA repair factors have the most direct relationship with HBV cccDNA formation; however, many complicated and indirect interactions with host machinery and HBV cccDNA formation are being discovered.

*PRPF31:* A spliceosome component, Pre-mRNA Processing Factor 31 (PRPF31), was discovered to be involved in cccDNA formation through a siRNA screening of 1,000 genes involved in DNA damage response and epigenetic and nucleic acid binding pathways [97]. The study demonstrated that PRPF31 associates with cccDNA and colocalizes with HBx in the nucleus, but whether PRPF31 interacts with nuclear rcDNA was not investigated in this study. When overexpressed together, PRPF31 and HBx, showed a marked increase in cccDNA formation that is not observed when either protein is overexpressed on their own. The exact mechanism of how a PRPF31-HBx complex enhances cccDNA formation remains unknown.

*CDK9:* The cyclin-dependent kinase (CDK) family proteins are largely responsible for the regulation of cell division or gene transcription [98]. CDK9 specifically is known to be involved in the transcription or replication of several viruses [99,100,101]. A CDK9-specific inhibitor, FIT-039, was able to reduce HBV replication in a dose-dependent manner without cytotoxicity [102]. Pan-inhibitors of the CDK family resulted in high toxicity and subsequent cell death. A time-to-addition treatment of infected HepG2-NTCP cells with FIT-039 showed the compound inhibited an early step in cell infection. Inhibition of NTCP was ruled out through an in vitro preS1 binding assay while Southern blot and qPCR analysis of cccDNA formation confirmed that FIT-039 reduced the level of cccDNA. HBV-infected chimeric mice showed a greater reduction of serum HBV DNA when treated with a combination of entecavir and FIT-039 compared to entecavir alone. These results imply that CDK9 may play a role in HBV cccDNA formation or stability.

*SAMHD1:* SAM and HD Domain Containing Deoxynucleoside Triphosphate Triphosphohydrolase 1 (SAMHD1) is stimulated by IFN and acts as a dNTPase to inhibit viral replication [103]. Vpx from the SIV_SMM_/HIV-2 lentiviruses was used to target and degrade SAMHD1 in infected HepG2-NTCP cells and resulted in a decrease of cccDNA levels [104]. Exogenous dNTPs were provided indicating the reduction of cccDNA levels was not dependent on dNTP levels. A CRISPR/Cas9 knockout of SAMHD1 resulted in a similar phenotype that was reversed by ectopic wild type SAMHD1 but failed with the expression of a mutated SAMHD1 lacking the nuclear localization signal. It has been reported that SAMHD1 can bind ssDNA and act as a scaffolding protein to promote both homologous recombination and DNA resection [105], indicating that the nuclear SAMHD1 may be involved in repairing rcDNA into cccDNA, but this study did not provide any direct evidence [104]. On the other hand, mRNA levels of APOBECs, MXA, and ISG20 were all observed to be higher in the SAMHD1 knockout cells when compared to the control, inferring a role of SAMHD1 in restricting host innate immunity. APOBEC3A/B, in particular, were shown to degrade cccDNA through cytidine deamination and apurinic/apyrimidinic site formation [72,106]. In this regard, SAMHD1 may also indirectly maintain the stability of cccDNA.

## 3. Summary and Perspectives

The picture being painted by these studies shows the formation of cccDNA relies heavily on the host DNA repair mechanisms and their surrounding pathways. The mystery of cccDNA formation is no longer as ambiguous as previously thought. Based on the findings, a proposed model of cccDNA formation would be: The mature rcDNA is deproteinated in the cytoplasm and transported into the nucleus, followed by nuclear uncoating of DP-rcDNA, which in turn activates ATR-Chk1 DNA damage response [9,17,24,25,64,76]. One copy of the (−) strand terminal redundant repeats is cleaved from the 5′ terminus by FEN1 or other nuclease(s) [73,82], followed by DNA resynthesis catalyzed by polymerase α and/or δ [36,79]. The (−) strand is then ligated shut to form CM-rcDNA [73]. Host DNA polymerases (α, δ, κ, η, etc.) then complete the (+) strand DNA for ligation [35,36,79]. Host DNA ligases (LIG1/3) and topoisomerases are involved in the ligation steps, while topoisomerases may have other functions beyond ligation [58,85] (Figure 4A). Although this cccDNA formation pathway seems possible, it is important to note that the participating host factors and the order of DNA repair reactions are likely to be appended and redefined as the research proceeds. It remains possible that these identified DNA intermediates (DP-rcDNA, CM-rcDNA) are minor or even byproducts of cccDNA formation, and major or additional intermediate(s) may exist and await further exploration. In addition, nearly the entirety of these studies failed to completely inhibit cccDNA formation when the host factors of interest were either knocked out or inhibited. This casts some doubt whether these are the essential factors for cccDNA formation and could suggest there are alternative host factors and/or pathways that serve redundant roles at each steps of the cccDNA formation pathway. In this regard, there is a plausible scenario of “all roads lead to Rome” during the conversion of rcDNA to cccDNA. In contrast, the involvement of NHEJ pathway in dslDNA-based cccDNA formation and integration seems more straightforward and well-documented (Figure 4B).

Yet another major cloud hanging over these studies on cccDNA formation is that most were performed using variants of the HepG2 or Huh7 cell line, derived from human hepatoma cells. The process of oncogenesis is well documented to have substantial alterations of cell functions, especially in DNA replication/repair and the cell cycle [107,108]. DNA repair pathways are closely regulated to the cell cycle, and therefore it is possible the DNA repair pathways of a hepatoma cell line are compromised and do not match wild-type DNA repair, leading to incomplete or inaccurate information. We believe it is pertinent, moving forward, to confirm these results and expand our search in more physiologically relevant human hepatocyte model systems. 

It is worth noting that a major hurdle of establishing a mouse model for HBV infection is the failure of cccDNA establishment in mouse hepatocytes, which is likely due to the lack of a human hepatocyte cellular factor(s) required for cccDNA formation [109,110,111]. However, it has been shown that HBV cccDNA could be made in an immortalized mouse hepatocyte cell line AML12 upon HBV stable transfection or infection [109,112,113], thus a better understanding of cccDNA formation in human hepatocytes and mouse AML12 cells will help to identify the missing factor(s) in mouse hepatocytes for cccDNA formation and facilitate the development of a mouse model for HBV infection.

As an essential step in HBV infection establishment and persistence, cccDNA formation offers multiple potential antiviral targets for developing novel HBV therapeutics. Based on the current state of knowledge, inhibiting the formation or stability of cccDNA intermediates such as DP-rcDNA and CM-rcDNA, and/or inhibiting the host DNA repair enzymes/factors involved in cccDNA formation, would be expected to further reduce cccDNA copy numbers and prevent virus spread in infected livers, ideally in combination with the available HBV replication inhibitors. In this regard, previous studies have identified compounds that reduce cccDNA formation via inhibiting DP-rcDNA production [67,114], and DNA repair targeted drugs are available in cancer therapy [115,116,117], which are candidates for antiviral assessment in cccDNA formation assays. Nonetheless, considering the longevity of cccDNA, the redundant activity of host DNA repair factors in cccDNA formation, and the nature of host targeting agents, the long-term efficacy and safety of cccDNA formation inhibitors should be closely monitored in future studies.

Taken together, it is envisioned that further investigations will delineate a coherent molecular pathway of HBV cccDNA formation, thereby providing new insights into cccDNA biology and novel antiviral targets for the development of therapeutics to cure hepatitis B.
